# Transcranial direct current stimulation in semantic variant of primary progressive aphasia: a state-of-the-art review

**DOI:** 10.3389/fnhum.2023.1219737

**Published:** 2023-11-08

**Authors:** Davide Norata, Francesco Motolese, Alessandro Magliozzi, Fabio Pilato, Vincenzo Di Lazzaro, Simona Luzzi, Fioravante Capone

**Affiliations:** ^1^Department of Medicine and Surgery, Unit of Neurology, Neurophysiology, Neurobiology and Psychiatry, Università Campus Bio-Medico di Roma, Rome, Italy; ^2^Neurological Clinic, Department of Experimental and Clinical Medicine (DIMSC), Marche Polytechnic University, Ancona, Italy; ^3^Fondazione Policlinico Universitario Campus Bio-Medico, Rome, Italy

**Keywords:** progressive aphasia, neurodegenerative disorders, dementia, semantic dementia, frontotemporal lobar degeneration, tDCS, non-invasive brain stimulation, neuromodulation

## Abstract

The semantic variant of primary progressive aphasia (svPPA), known also as “semantic dementia (SD),” is a neurodegenerative disorder that pertains to the frontotemporal lobar degeneration clinical syndromes. There is currently no approved pharmacological therapy for all frontotemporal dementia variants. Transcranial direct current stimulation (tDCS) is a promising non-invasive brain stimulation technique capable of modulating cortical excitability through a sub-threshold shift in neuronal resting potential. This technique has previously been applied as adjunct treatment in Alzheimer’s disease, while data for frontotemporal dementia are controversial. In this scoped review, we summarize and critically appraise the currently available evidence regarding the use of tDCS for improving performance in naming and/or matching tasks in patients with svPPA. Clinical trials addressing this topic were identified through MEDLINE (accessed by PubMed) and Web of Science, as of November 2022, week 3. Clinical trials have been unable to show a significant benefit of tDCS in enhancing semantic performance in svPPA patients. The heterogeneity of the studies available in the literature might be a possible explanation. Nevertheless, the results of these studies are promising and may offer valuable insights into methodological differences and overlaps, raising interest among researchers in identifying new non-pharmacological strategies for treating svPPA patients. Further studies are therefore warranted to investigate the potential therapeutic role of tDCS in svPPA.

## 1. Introduction

In the 1980s, the recognition of clinical entities distinct from Alzheimer’s disease led to the identification of primary progressive aphasias (PPAs) (Mesulam, 1987), neurodegenerative disorders characterized by early and prominent speech or language impairment with relative sparing—at least in the mild stage—of other cognitive domains, such as memory or behavior. Although other symptoms may present in the later stages of PPA, language impairment remains the most prominent feature (Gorno-Tempini et al., 2011).

Three variants of PPA are recognized. While the *logopenic variant* is largely considered as an atypical variant of Alzheimer’s disease, the *nonfluent*/*agrammatic variant* and the *semantic variant* (svPPA) are classified as frontotemporal dementia (FTD) syndromes because of their association with frontotemporal lobar degeneration pathology (Mendez, 2019). Differentiating between PPA subvariants can be challenging due to overlapping clinical features. However, an accurate diagnosis is crucial for optimal clinical management and targeted interventions.

According to the current diagnostic criteria (Pick, 1892; Gorno-Tempini et al., 2011), the first diagnostic step requires proving that the primary clinical feature is language impairment, without other clinical-neuropsychological features that may indicate an alternative diagnosis (Table 1). The current criteria also include a set of mandatory core features and supporting features that allow clinically accurate identification of the three main PPA variants (Table 2).

**Table 1:** Root criteria for the diagnosis of primary progressive aphasia (PPA).

Inclusion criteria
1. Most prominent clinical feature is difficulty with language
2. These deficits are the principal cause of impaired daily living activities
3. Aphasia should be the most prominent deficit at symptom onset and for the initial phase of the disease

Exclusion criteria
1. Pattern of deficits is better accounted for by other nondegenerative nervous system or medical disorders
2. Cognitive disturbance is better accounted for by a psychiatric diagnosis
3. Prominent initial episodic memory, visual memory, and visuo-perceptual impairments
4. Prominent, initial behavioral disturbance

Modified from Gorno-Tempini et al. (2011).

**Table 2:** Criteria for variants of primary progressive aphasia (PPA).

	Nonfluent/agrammatic variant PPA	Logopenic variant PPA	Semantic variant PPA
Core features	≥ 1 *of the following core features*: Agrammatism in language production Effortful, halting speech with inconsistent speech sound errors (apraxia of speech)	*Both of the following core features*: Impaired single-word retrieval in spontaneous speech and naming Impaired repetition of sentences and phrases	*Both of the following core features*: Impaired confrontation naming Impaired single-word comprehension
Supportive features	≥ 2 *of the following*: Impaired comprehension of syntactically complex sentences Spared single-word comprehension Spared object knowledge	≥ 3 *of the following*: Speech (phonologic) errors in spontaneous speech and naming Spared single-word comprehension and object knowledge Speared motor speech Absence of frank agrammatism	≥ 3 *of the following*: Impaired object knowledge, particularly for low-frequency or low-familiarity items Surface dyslexia or dysgraphia Speared repetition Speared speech production (grammar and motor speech)

Modified from Gorno-Tempini et al. (2011).

After Pick’s 19th-century description of a progressive language disorder associated with frontotemporal atrophy (Pick, 1892), and Warrington’s, 1975 paper about the selective impairment of semantic memory in three patients with anomia (Warrington, 1975), in 1989 Snowden proposed the term “semantic dementia” (SD) (Snowden et al., 1989), defining for the first time a condition that has continued to fascinate behavioral neurologists in the 21st century. Despite some authors defining svPPA as the purely linguistic form of SD (Adlam et al., 2006), using the term “semantic dementia” for indicating the progressive impairment of multi-modal semantic representations (Hodges et al., 1992; Mesulam, 2001, 2003), in this paper the two terms are used interchangeably (Gorno-Tempini et al., 2011).

Although research on SD prevalence is lacking, it is expected to account for one-fourth to one-third of FTD cases (Hodges and Patterson, 2007; Landin-Romero et al., 2016), i.e., 2.5–7.3 per 100,000 people (Onyike and Diehl-Schmid, 2013; Landin-Romero et al., 2016). SD usually manifests before the age of 65, but one-fourth of cases may occur after the age of 70 (Hodges et al., 2010). It severely impairs patients’ communication abilities, having a significant influence on their familial and socio-professional life (Hodges et al., 2010). The median survival is on the order of 10 to 13 years, which is longer than is usually associated with FTD (Hodges et al., 2010).

From a pathological standpoint, the regions primarily affected by SD-related neurodegeneration are the temporal pole and the anterior temporal lobe (ATL) of both hemispheres (Gorno-Tempini et al., 2011; Mesulam et al., 2014), with approximately 70% of patients presenting with major left-sided involvement and 30% presenting with predominant right-sided involvement (Hodges et al., 2010; Kumfor et al., 2016).

Overall, SD patients experience a progressive and severe loss of conceptual knowledge, with the main deficit involving word meaning (Gorno-Tempini et al., 2011; Mesulam et al., 2014). This results in anomia, impaired word comprehension and fluent but content-empty speech (Davies et al., 2005), and with preserved grammar and speech articulation (Mesulam et al., 2014). Furthermore, SD can affect face recognition (Snowden et al., 2004; Luzzi et al., 2017), object feature attribution (Garrard and Carroll, 2006), sound-picture matching (Bozeat et al., 2000), object-use matching (Corbett et al., 2009), and arithmetic knowledge (Luzzi et al., 2013). The clinical presentation varies according to the side of the brain that is mainly affected by the degenerative process. The most noticeable symptom of left-sided SD is the involvement of language, with anomia and loss of single-word meaning. In right-sided SD, the prominent impairment is associative prosopagnosia (i.e., the inability to recognize familiar people’s faces), together with the inability to recognize animals and items.

Previous studies have found a link between ATL gray matter loss and impairment in a variety of semantic tasks [as picture naming (Rosen et al., 2002; McMillan et al., 2004) and word-picture association (Mummery et al., 2000)]. As a result of ATL degeneration, functional connectivity between brain areas in the frontal, temporal, parietal, and occipital lobes, including visual and auditory association cortices, is compromised (Guo et al., 2013).

The loss of connectivity has been evaluated structurally as a decrease of white matter volume in the left temporal lobe, periventricular white matter, and the corpus callosum (Good et al., 2002), as well as damage to white matter tracts such as the inferior longitudinal fasciculus and the uncinate fasciculus (Rohrer et al., 2010). Furthermore, even if the Papez circuit is likely implicated in PPA pathogenesis, the mammillary bodies and the body and tail of the hippocampus are frequently spared, and this might explain why the episodic memory is spared (Tan et al., 2014). Cortical hypometabolism of the ATL cortex—and occasionally of the subgenual area and the right anterior cingulate cortex –is a valuable imaging hallmark useful for the diagnosis (La Joie et al., 2014).

There is no approved treatment for svPPA and the other PPA variants because speech therapy protocols have not been validated and pharmaceutical treatments have not demonstrated significant results (Vercelletto et al., 2011; Boxer et al., 2013; Monti et al., 2013). Non-pharmacological approaches focused on non-invasive brain stimulation (NIBS) might then have a role in disease treatment and diagnosis (Di Lazzaro et al., 2021). Indeed, it is known that neurodegenerative conditions of different etiologies have their own “neurophysiological signature” in terms of selective involvement of neural circuits. This represents the rationale for the therapeutical application of NIBS techniques such as repetitive transcranial magnetic stimulation (Lambon Ralph, 2014).

Clinically, svPPA is primarily characterized by a significant impairment in a particular cognitive function, specifically semantic knowledge. This feature is accompanied, uniquely in this variant and notably in the initial stages of the disease, by a corresponding “neuroanatomical signature,” denoting the engagement of discrete brain regions (mainly the ATL and the temporal pole). This SD uniqueness underscores the significance of a comprehensive study, as it holds promising implications for unraveling the underlying neural mechanisms associated with language processing and semantic memory (Deakin et al., 2004).

Transcranial direct current stimulation (tDCS) is one of the most widely employed NIBS techniques, characterized by numerous advantages (e.g., cost, portability, comfort, possibility of delivering during rehabilitation). It consists of a weak electric current (with an average intensity of 1–2 mA) delivered between two electrodes placed on separate areas of the scalp (Elder and Taylor, 2014). The electric current produces a polarization gradient between electrodes that modulates cortical excitability through a sub-threshold alteration of the resting membrane potential (Ulm et al., 2015). Although tDCS has been used for several years in neurological, otorhinolaryngological, and psychiatric research, a 2017 comprehensive review highlighted that we currently have a sufficient level of evidence to provide recommendations for employing tDCS in just a few disorders (i.e., chronic neuropathic pain due to spinal cord lesion, fibromyalgia, tinnitus, depression, and addiction/craving syndromes) (Lefaucheur et al., 2017). According to that review, for the rest of the conditions on which it has been tried, tDCS cannot yet be the subject of therapeutic recommendations because of the often-inconsistent trial results. This discrepancy would likely be due to differences in the protocols used, such as in current intensity, number and timing of stimulation sessions, placement, shape and size of electrodes (Lefaucheur et al., 2017).

The goal of this study is to describe and critically evaluate the current available evidence on tDCS-based therapy for improving language in patients with svPPA and suggest implications for the future research.

## 2. Methods

This scoped review adopted a systematic approach in the processes of research question selection, search and selection of literature.

Clinical trials published up to November 2022 (week 3) and addressing the topic were identified using Web of Science and MEDLINE accessed by PubMed. We developed subject-specific search strategies for each academic database, as follows.

Web of Science search strategy: [TS = (semantic dementia)] OR [TS = (svPPA)] OR [TS = (semantic variant PPA)] OR [TS = (semantic variant primary progressive aphasia)] AND [TS = (tDCS)] OR [TS = (transcranial direct current stimulation)].

MEDLINE search strategy: [semantic dementia (Title/Abstract)] OR [svPPA (Title/Abstract)] OR [semantic variant primary progressive aphasia (Title/Abstract)] OR [frontotemporal dementia (Title/Abstract)] AND [tDCS (Title/Abstract)] OR [electrical brain stimulation (Title/Abstract)] OR [transcranial direct current stimulation (Title/Abstract)].

The literature selection and exclusion were conducted by a single researcher, based on the PICOS (Patient Intervention Comparison Outcome Study design) of the PRISMA protocol (Moher et al., 2009). The present review included: (1) studies conducted on svPPA patients, (2) studies evaluating the clinical effects of tDCS, (3) studies with an experimental design, and (4) studies written in English. The exclusion criteria were: qualitative studies, case reports, unpublished papers and articles written in other languages. Duplicated publications were excluded by comparing titles and abstracts. Moreover, publications not related to the study topic were excluded.

Although the methodological footprint employed by the present study resembles that of a systematic review, it endeavors to address a highly specific question, necessitating the inclusion of articles that require qualitative analysis of figures and raw data. Furthermore, this study was not prospectively registered in any of the register for systematic reviews. For these two reasons, the study has been conducted as a scoped review.

As a result, the final six publications were reviewed.

Supplementary Figure S1 shows the flowchart of the study selection process, according to the PRISMA protocol.

## 3. Results

Table 3 summarizes the main features of published data on tDCS in svPPA patients, as described in this review.

**Table 3:** Summary of the results of the reviewed trials.

Study, year	svPPA population	Age, mean ± st. dev.	Protocol design	tDCS approach	tDCS montage
Teichmann et al. (2016)	12	66.8 ± 2.1	Randomized sham-controlled crossover design on svPPA patients	20 min tDCS (1.59 mA), single session	2 conditions: A-anode over left temporal pole (FT7-FT9) and cathode on contralateral supra-orbital region (AF8); B-cathode in right temporal pole (FT8–FT10) and anode in contralateral supraorbital region (AF7)
Hung et al. (2017)	3	67.3 ± 8.7	Case series pre-post design on patients with all-PPA variant	30 min tDCS (1.5 mA), 10 sessions over 2 weeks	Anode over left temporoparietal region (P3), cathode centered on forehead
Roncero et al. (2017)	2	63.5 ± 7.5	Randomized, within subject crossover design with AD and all-PPA variant patients	30 min tDCS (2 mA), 10 sessions over 18 days	Anode over left inferior temporoparietal region (P3), cathode on right fronto-orbital area
Tsapkini et al. (2018)	10	68.6 ± 5.2	Randomized, within-subject crossover design on patients with all-PPA variant	20 min tDCS (2 mA), 15 sessions	Anode over left inferior frontal lobe (F7), cathode on right cheek
Ficek et al. (2018)	8	Not clarified in the text	Randomized, within-subject crossover design on patients with all-PPA variant	20 min tDCS (2 mA), 15 sessions	Anode over left inferior frontal lobe (F7), cathode on right cheek
Roncero et al. (2019)	4	61.8 ± 7.2	Randomized, within subject crossover design on patients with all-PPA variant	30 min tDCS (2 mA), 10 sessions over 21 days	2 conditions: A-anode over left parieto-temporal region (TP9), cathode on right fronto-orbital area; B-anode over DLPFC (F3), cathode over right deltoid muscle

Sham tDCS	Follow-up, weeks	Language training	Primary behavioral outcome measure	Oral naming outcome measures	Written naming outcome measures	Results in svPPA patients
Yes	None	None	Accuracy of a “find the intruder” test among items, presented verbally or visually. No naming accuracy test	None	None	In the verbal modality both left-excitatory and right-inhibitory tDCS improved performance in a “find the intruder” task. No differences between left and right tDCS effects. No effects in visual modality
None	24	Semantic treatment approach, during tDCS	Naming accuracy: percentage of correct answers	Naming of trained and untrained nouns	None	Not clarified in the text. A figure reporting raw individual data seems to show a significant effect of tDCS on svPPA patients. The authors report that patients most likely to see a sustained benefit from tDCS were all diagnosed with semantic variant PPA
Yes	4	Picture naming training, during tDCS	Naming accuracy: percentage of correct picture naming	Naming of trained and untrained nouns	None	Not clarified in the text. Two figures reporting raw individual data, seem to show a significant effect of tDCS on naming of trained and untrained items
Yes	2 and 8	Oral and written naming/spelling therapy, during tDCS	Written naming spelling accuracy: percentage of correct letters from treated and untreated words	Naming of trained and untrained nouns	Written naming/spelling of trained and untrained word	The spelling of trained and untrained items improved without any significant difference in both group (tDCS + language therapy and sham + language therapy)
Yes	None	Oral and written naming/spelling therapy, during tDCS	Written naming spelling accuracy: percentage of correct letters from treated and untreated words	None	Written naming/spelling accuracy of trained and untrained words	Not clarified in the text. A figure seems to show a significant effect of tDCS on svPPA patients, indicating that data were collected, even if not presented in a systematic way in the text
Yes	2 and 8	Picture naming training, during tDCS	Naming accuracy: percentage of correct picture naming	Naming of trained and untrained nouns	None	Without conducting formal analysis, the authors simply present raw individual data about naming accuracy of untrained items before and immediately after the 10 sessions of parieto-temporal tDCS, showing that only one svPPA subject improved

st. dev., standard deviation; svPPA, semantic variant of primary progressive aphasia; tDCS, transcranial direct current stimulation; DLPFC, dorsolateral prefrontal cortex. The electrode locations are also expressed using the International 10-10 system.

So far, only one trial specifically addressed the efficacy of tDCS in improving semantic performance (evaluated as picture-to-picture and word-to-word matching tests) in svPPA patients (Teichmann et al., 2016) while the other available data originate from trials designed to investigate the effects of tDCS in different degenerative speech diseases, including svPPA (Hung et al., 2017; Roncero et al., 2017; Ficek et al., 2018; Tsapkini et al., 2018). For the purpose of the present review, we used the specific subgroup analysis about the semantic variant reported in one study (Tsapkini et al., 2018), while for the others (Hung et al., 2017; Roncero et al., 2017; Ficek et al., 2018) we addressed the raw data explicitly related to svPPA patient, or plots for distinct subgroups, as reported by the authors.

Teichmann et al. (2016) conducted a monocentric, randomized, sham-controlled, crossover trial for evaluating the efficacy of tDCS in improving svPPA patients’ performance in semantic word-to-word and picture-to-picture matching. SvPPA diagnosis was based on neuropsychological testing, Gorno-Tempini’s criteria, and metabolic imaging-supported evidence (i.e., PET-FDG). The authors found that a single 20 min session of tDCS (1.59 mA) enhanced performance in a “find the intruder”-type task when compared to sham stimulation (Table 3; Teichmann et al., 2016). Twelve participants were enrolled and underwent three stimulation sessions each: left excitatory tDCS (with the anode over the left temporal pole and the cathode over the contralateral supra-orbital region), right inhibitory tDCS (with the cathode over the right temporal pole and the anode over the contralateral supraorbital region), and sham. The study cohort’s average age was 66.8 years, and 33% were female. Cognitive assessments were carried out at baseline and immediately after the tDCS session, with no further follow-up or language training. The primary efficacy outcome of improved performance in the word-to-word matching task occurred in both left-excitatory tDCS (baseline 33% ± 29 correct, post-tDCS 48% ± 23 correct; *F* = 5.5, *p* = 0.029) and right-inhibitory tDCS (baseline 28% ± 26 correct, post-tDCS 43% ± 32 correct; *F* = 6.5, *p* = 0.027), whereas sham stimulation had no effect on performance (baseline 34% ± 27 correct, post-sham 31% ± 24 correct; *F* < 1). Moreover, analyses on reaction times revealed that right-inhibitory tDCS speeded post-stimulation reactions for the “living” category of presented items [baseline (3,575 ms ± 749, post-tDCS 3,269 ms ± 650, *F* = 11.0, *p* = 0.007)] (Teichmann et al., 2016).

Hung et al. (2017) designed a simple clinical trial without a control group that included both pre- and post-tDCS assessments employing an oral naming task. Diagnoses were based on clinical criteria. In addition to a semantic training strategy based on Reilly et al. (2016) research, they administered 10 sessions of 30 min tDCS (1.5 mA) over 2 weeks, with the anode over the left inferior temporoparietal area and the cathode centered on the forehead. A 6 months follow-up visit with a new cognitive testing was also reported. A total of 5 individuals with semantic or logopenic PPA or Alzheimer’s disease were recruited in the research. The svPPA cohort contained three patients with an average age of 67.3 years of which one was female. This paper does not expressly disclose results in the subset of svPPA individuals, but it does contain an image reporting raw individual data that appears to demonstrate a significant effect of tDCS on naming performance. Furthermore, in the discussion session, the authors note that svPPA patients were most likely to experience a sustained benefit from tDCS (Hung et al., 2017).

Roncero et al. (2017) presented a randomized, crossover-design, sham controlled clinical trial designed to study the efficacy of tDCS in patients with Alzheimer’s disease or PPA. Diagnoses were made according to neuropsychological and language testing, clinical criteria, and PET-FDG findings. In this study, the anode was placed over the left inferior temporoparietal region, while the cathode was placed over the right fronto-orbital region. The experimental phase consisted of ten 30 min tDCS (2 mA) treatments for 18 days in combination with a picture-naming training, compared with sham stimulation plus the same training. Two svPPA patients were enrolled among a total of 10 participants. The svPPA-mean group’s age was 63.5 years, and both patients were female. Two figures in the text showing raw individual data seem to demonstrate a measurable impact of tDCS on naming of trained and untrained items, although no explicit results for patients with svPPA are mentioned in the manuscript (Roncero et al., 2017).

Tsapkini et al. (2018) conducted a randomized, within-subject, crossover trial on patients with primary progressive aphasia (all variants), comparing the effect of 15 sessions of 20 min tDCS (2 mA, with the anode over the left inferior frontal gyrus and the cathode on the right cheek) in addition to speech therapy, with sham stimulation and speech therapy. Diagnoses were based on Gorno-Tempini criteria. A total of 36 patients were enrolled; among them, 10 suffered from svPPA, with a mean age of 68.6 years (five females). The cognitive evaluations were performed at baseline, immediately after the tDCS sessions, after 2 weeks, and after 2 months from the last session, and focused especially on written naming and spelling accuracy. In the subgroup analysis conducted on svPPA patients, they did not find any significant effect of tDCS as add-on to language training, at each timepoint (trained items effect size = 0.07, 95% CI 0.70–20.56, *p* = 0.793; untrained items effect size = 0.36, 95% CI 2.66–21.94, *p* = 0.466) (Tsapkini et al., 2018).

In 2018, the same research group published another paper presenting a subgroup analysis of their trial. In this study, they employed the same stimulation paradigm (15 sessions of 20 min anodal 2 mA tDCS over the left inferior frontal gyrus versus sham stimulation, in addition to language therapy) (Ficek et al., 2018), but the patient’s accurate diagnosis was determined not only through neuropsychological/language testing and clinical criteria but also using structural brain imaging, namely magnetic resonance imaging (MRI). The semantic variant of PPA’s cohort included 8 individuals (descriptive characteristics of subgroups, such as average age and sex, are not reported in the publication). Although results for patients with svPPA are neither explicitly nor systematically presented, a figure published within the paper seems to demonstrate a significant impact of tDCS on writing accuracy following 15 stimulations (Ficek et al., 2018).

Roncero et al. (2019) published another paper in 2019 focusing on individuals with all-variant PPA. The study was designed as a randomized, within-subject sham-controlled crossover trial, assessing the effect of 10 sessions of 30 min tDCS at 2 mA, coupled with language training for a picture-naming task. A formal diagnosis of PPA was established through neuroimaging scans (e.g., FDG PET, MRI) and neuropsychological testing. The efficacy of sham tDCS was compared to two active tDCS configurations: parieto-temporal anodal stimulation, with the anode positioned over TP9 and cathode on the right fronto-orbital area; and dorsolateral prefrontal cortex (DLPFC) stimulation, with the anode placed over left DLPFC (F3) and the cathode over the right deltoid muscle. Follow-up assessments were conducted at 2 weeks and 2 months after the final tDCS session. Although no formal analysis was performed on the four sv-PPA patients (mean age 61.8 years, one female) included out a total of 27 enrolled individuals, the authors observed an immediate improvement in naming accuracy of untrained items only in one svPPA participant following the 10 sessions of parieto-temporal tDCS (Roncero et al., 2019).

## 4. Discussion

To date, few data have been published about the effects of tDCS on cognitive functions in svPPA patients. These data are characterized by significant methodological heterogeneity as detailed below.

### 4.1. Anodal vs. cathodal tDCS

TDCS can deliver electric stimulations in two different ways: anodal and cathodal stimulation. Anodal stimulation (i.e., placing the anode over the targeted area) is supposed to shift the membrane resting potential of nearby neurons, increasing the neural excitability of the stimulated area (excitatory effects). Cathodal stimulation (with the cathode positioned over the targeted region) lowers the membrane potential away from the firing threshold, reducing regional excitability (inhibitory effects) (Sanches et al., 2019).

Several studies have demonstrated the effects of NIBS techniques in patients with post-stroke aphasia and ischemic lesions sited in the left hemisphere. Specifically, tDCS has been applied to improve functional recovery in aphasic stroke patients, through the promotion of neural plasticity. Language-related networks, which may be modulated using NIBS, have nodes in both hemispheres, because transcallosal connections (Park et al., 2008; Kaplan et al., 2010) connect the left and right homotopic areas, according to the notion of inter-hemispheric communication (Ferbert et al., 1992). Non-invasive brain stimulation has been used to treat left hemisphere stroke aphasia in three ways (Fregni and Pascual-Leone, 2007; Hamilton et al., 2011): (A) excitatory stimulation (anodal tDCS) on left hemisphere language areas, to activate perilesional regions and reactivate language processes; (B) inhibitory stimulation (cathodal tDCS) on right hemisphere, to reduce trans-callosal inhibition; (C) excitatory stimulation (anodal tDCS) on right hemisphere, to activate potential language networks sited there. Recent evidence suggests that tDCS approaches should be chosen depending on the structural reserve of the injured hemisphere. If such reserve is inadequate, the unaffected hemisphere will try to compensate for the functional deficit, and it should need support (stimulating tDCS). If, on the other hand, the stroke hemisphere has a sufficient amount of structural reserve, the unaffected hemisphere would exert a disproportionate inhibition on the contralateral, and should be suppressed to promote recovery [bimodal balance recovery model; for a comprehensive review see (Di Pino et al., 2014; Di Pino and Di Lazzaro, 2020)].

The anodal tDCS on left hemisphere and the cathodal tDCS on right hemisphere proved to be more promising in determining clinical outcomes on language disabilities (Monti et al., 2008; Baker et al., 2010; Fiori et al., 2011). Researchers observed transient beneficial effects with single tDCS-session, as well as longer-lasting effects (>6 months) with periodic stimulations, which most likely promoted neuroplasticity (Naeser et al., 2005, 2011).

Regarding svPPA patients, two studies were conducted on anodal stimulation of the left inferior frontal lobe, with the anode placed on F7 (according to the international EEG 10–20 system) and the cathode on the right cheek (Ficek et al., 2018; Tsapkini et al., 2018), and two studies carried out the excitatory tDCS of the left temporoparietal region, with the anode placed on P3 (according to the international EEG 10–20 system) and cathode centered on forehead (Hung et al., 2017) or sited on the right fronto-orbital area (Roncero et al., 2017).

Teichmann et al. (2016) investigated the impact of more than one tDCS modality in svPPA patients. For this reason, their protocol was designed to compare the anodal excitatory stimulation of left ATL with the cathodal inhibition of right ATL, finding no differences in the primary endpoint. The main limitation of that study is that it failed to take into account an intrinsic feature of the semantic variant of PPA, namely that 30% of patients might have predominant right-ATL involvement (Hodges et al., 2010; Kumfor et al., 2016), that might exacerbate symptoms with a right-inhibitory tDCS.

### 4.2. Stimulation sites

**Figure 1:**
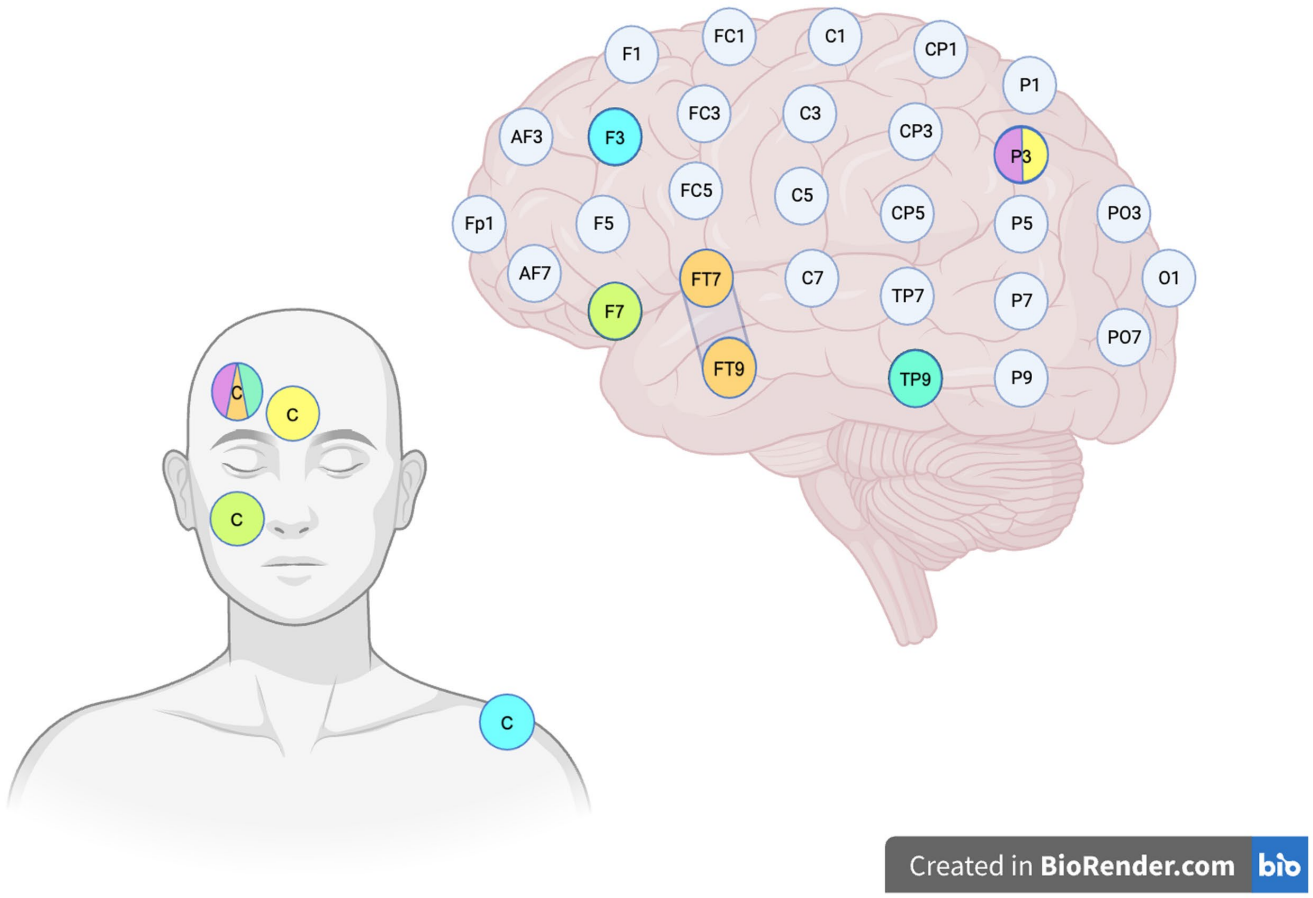
Stimulation sites. The image illustrates the various tDCS configurations employed in the reviewed studies, based on the EEG International 10-10 system. The color legend is set as follows: **Orange:** Teichmann et al. (2016) left anodal configuration, where the anode is placed over the left temporal pole and the cathode (C) is positioned on the contralateral supraorbital region. The second condition, which is not depicted in the figure, is designed with cathode placed over the right FT8–FT10 and the anode on the left supraorbital region. **Green:** configuration employed by Tsapkini et al. (2018) and Ficek et al. (2018), with the anode over the left inferior frontal lobe and the cathode (C) on the right cheek. **Yellow:** tDCS montage used by Hung et al. (2017), with the anode positioned over the left parieto-temporal region and the cathode (C) centered on the forehead. **Lavender:** configuration utilized by Roncero et al. (2017), with the anode over the left parieto-temporal region and the cathode placed on the right fronto-orbital region. **Aquamarine:** Roncero et al. (2019) inferior parieto-temporal anodal stimulation, with the anode positioned over TP9 and the cathode on the right fronto-orbital region. **Cyan:** Roncero et al. (2019) DLPFC stimulation, with the anode over F3 and the cathode sited over the right deltoid muscle.

Table 4 provides a brief overview of the hypothetical objectives linked to each tDCS setup employed in the reviewed studies. Figure 1 shows the different tDCS configurations.

**Table 4:** The rationale for each tDCS setup employed by the reviewed studies.

Authors, year	tDCS configuration overview	Rationale for the chosen target
Teichmann et al. (2016)	Anodal tDCS over the left temporal pole	Activating the verbal semantic network to enhance naming and matching
	Cathodal tDCS over the right temporal pole (reducing the interhemispheric transcallosal inhibition)	
Hung et al. (2017)	Anodal tDCS over the left parieto-temporal region	Activating the posterior superior temporal cortex and the temporoparietal hub for semantic control processing to enhance naming
Roncero et al. (2017)		
Tsapkini et al. (2018)	Anodal tDCS over the left IFG	Activating the main language hub for enhancing writing ability, orthographic long-term memory, and phoneme-to-grapheme conversion
Ficek et al. (2018)		
Roncero et al. (2019)	Anodal tDCS over the inferior left parieto-temporal region	Activating the superior temporal gyrus, the angular gyrus, and parietal areas, crucial for fact retrieval and semantic control processing to enhance naming
	Anodal tDCS over the left DLPFC	Activating areas involved in attention, executive functions, and working memory, connected to language networks

tDCS, transcranial direct current stimulation; IFG, inferior frontal gyrus; DLPFC, dorso-lateral prefrontal cortex.

One of the Teichmann et al. (2016) study’s strengths is that it targeted the left or right temporal pole, which is the more clearly involved area in svPPA/SD pathogenesis (Gorno-Tempini et al., 2011; Mesulam et al., 2014). However, we have to acknowledge that PPA is often diagnosed at late stage, where cortical atrophy due to aging might impair the effectiveness of neuromodulatory techniques.

By contrast, both Hung et al. (2017) and Roncero et al. (2017) trials aimed to stimulate the left inferior temporoparietal region, that has been previously described as a critical hub region for semantic control processing, particularly for naming (Koenigs et al., 2009; Price et al., 2016; Reilly et al., 2016).

In their paper, Roncero et al. (2019) employed two distinct active tDCS configurations, one targeting the inferior left parietotemporal area (TP9) and the other stimulating the left DLPFC. Recent research has identified the white matter of the left parietotemporal region, which encompasses the superior temporal gyrus, the angular gyrus, and parietal areas, as an additional network that plays a significant role in observed fact retrieval and arithmetic fact retrieval (Smaczny et al., 2023). In NIBS clinical trials on patients with mild cognitive impairment or dementia due to Alzheimer’s disease, the left DLPFC is frequently selected as the cortical target due to its crucial role in cognitive functions such as attention, executive functions, and working memory (Lambon Ralph, 2014; Lara and Wallis, 2015).

Finally, both Tsapkini et al. (2018) and Ficek et al. (2018) targeted the left IFG (inferior frontal gyrus), one of the main language hubs, involved in the writing ability, the orthographic long-term memory, and the phoneme-to-grapheme conversion (Purcell et al., 2011; Rapp and Dufor, 2011; Planton et al., 2013; DeMarco et al., 2017). The IFG is involved in all-variant PPA: in *non*-*fluent*/*agrammatic variant*, the left IFG is the primary site of cortical atrophy; *in logopenic variant*, atrophy is especially noticeable in the left supramarginal and angular gyri, which the IFG connects via the dorsal language stream (Hickok and Poeppel, 2004), the superior longitudinal fasciculus III, or the arcuate fasciculus (Catani et al., 2003); *in semantic variant*, the ATL is the major atrophy center, connected to the IFG through the ventral language stream, which includes the uncinate fasciculus (Hickok and Poeppel, 2004).

Furthermore, it is worth pointing out that in the studies conducted by Teichmann et al. (2016) and Roncero et al. (2019), the placement of electrodes was determined based on a brain MRI of the subjects, allowing for more precise estimation of the targeted coordinates.

### 4.3. TDCS intensity

As shown in Table 3, the reviewed studies used a defined current intensity of tDCS, but this differed between protocols.

While the majority used an arbitrary current intensity of 2 mA, Hung et al. (2017) used 1.5 mA (again an arbitrarily chosen value).

On the other hand, Teichmann et al. (2016) used 1.59 mA, calculated starting from a current density of 0.06 mA/cm^2^, as previously used in post-stroke aphasia or PPA studies with larger leads (Monti et al., 2008; Tsapkini et al., 2014). The purpose of this adjustment is to apply the same current intensity regardless of electrode shape and size.

### 4.4. Behavioral outcome measures

Regarding the discrepancies in the cognitive outcome chosen to measure changes in semantic tasks such as naming and matching, it is evident that the only study that has specifically addressed svPPA patients is that of Teichmann et al. (2016), with its “find the intruder”-type tasks. It consisted of a test based on the famous Pyramid and Palm Trees Test (Howard and Patterson, 1992), and participants had to select which of the two given items was related to the test item as accurately and promptly as possible. The items might be written words or pictures of either live or non-living beings.

The studies examining patients with various variants of progressive aphasia opted to evaluate production tasks, such as naming/writing accuracy. More specifically, Hung et al. (2017) and Roncero et al. (2017, 2019) used the naming of trained and untrained nouns (i.e., a verbal task), while both Tsapkini et al. (2018) and Ficek et al. (2018) measured letter accuracy of treated and untreated written words as the main outcome measure of their study. These outcomes were adopted because oral or written spelling accuracy is compromised in all degenerative forms of language impairment, even if it is not exclusive of svPPA patients.

### 4.5. Strengths and limitations of the present study

The main, implicit advantage of studying svPPA is the opportunity to observe a progressive aphasia that affects a well-defined brain region (the ATL). It follows that it might pave the way for the development of an experimental model of degenerative aphasias and their NIBS-based treatment.

However, as previously detailed, there are major differences among trials, regarding the study population (including different forms of dementia often without a biological diagnosis), time from disease onset (or rather, the lack of information on the progression of the disease for each patient), tDCS setups and intensities, stimulation sites, and outcome measures.

This methodological heterogeneity hinders the ability to draw definitive conclusions on the efficacy of tDCS-based treatments in svPPA patients.

Another critical limitation of this study is the significant impact of small sample sizes of svPPA patients on the cited results. Furthermore, the majority of these studies either do not specifically target svPPA patients or lack treatments tailored to the brain areas primarily affected by this disease. The insufficient sample size reduces the likelihood of obtaining statistically significant results with adequate statistical power, leading to an increased risk of false negatives and reduced result reliability. Considering the aforementioned methodological heterogeneity, this review cannot overcome the bias of inadequate sample size through meta-analysis. As a result, the conclusions that can be drawn from this study are considerably restricted.

## 5. Conclusion and future directions

With the exception of a single trial (Tsapkini et al., 2018) and major concerns about the methodological heterogeneity of the included papers, all of the findings of the selected studies suggest the improvement of the semantic performance of patients with svPPA following non-invasive electrical stimulation.

If the goal is to develop the most effective non-invasive treatment tailored to each patient, the aforementioned concerns need to be taken into account. To determine the most optimal tDCS arrangement, appropriate cognitive tests and neurophysiological evaluations (e.g., quantitative electroencephalography, QEEG) (Livint Popa et al., 2020) would be useful tools to identify the involved networks and differentiate right from left SD patients with high specificity. Furthermore, obtaining current intensity values from a safe and effective predefined value of current density, in combination with the selection of an appropriate stimulation site and a homogenous outcome variable, would allow for experiment standardization across centers despite the use of different equipment.

Because svPPA is a very rare disease, with huge recruitment difficulties and the added challenge of being able to intervene with NIBS only in the mild stage of the disease, most authors included svPPA patients in tDCS-studies involving subjects with other forms of frontotemporal lobar degeneration clinical syndromes and even patients with Alzheimer’s disease. Multi-center trials (or, at the at least, standardizing monocentric studies) could represent a reasonable next strategy. These would allow researchers to remain focused on a single nosological entity. A greater specificity may represent a promising way to achieve more significant and reproducible results that may be integrated in future guidelines.

## Data availability statement

The original contributions presented in the study are included in the article/Supplementary material, further inquiries can be directed to the corresponding author.

## Author contributions

FM, FC, and DN: conceptualization. FC and FM: methodology. DN: software, investigation, resources, data curation, visualization, and project administration. DN and SL: writing—original draft preparation. FC, FP, AM, and FM: writing—review and editing. VL, FP, FC, and SL: supervision. All authors contributed to the article and approved the submitted version.
