# Supplementary material for: Transcranial direct current stimulation in semantic variant of primary progressive aphasia: a state-of-the-art review
Source: Front Hum Neurosci. 2023 Nov 8;17:1219737. doi: 10.3389/fnhum.2023.1219737 (PMC10663282; doi:10.3389/fnhum.2023.1219737)
Supplement: Supplementary file 1 [file Image_1.pdf]

Supplementary Material

1 Supplementary Figures

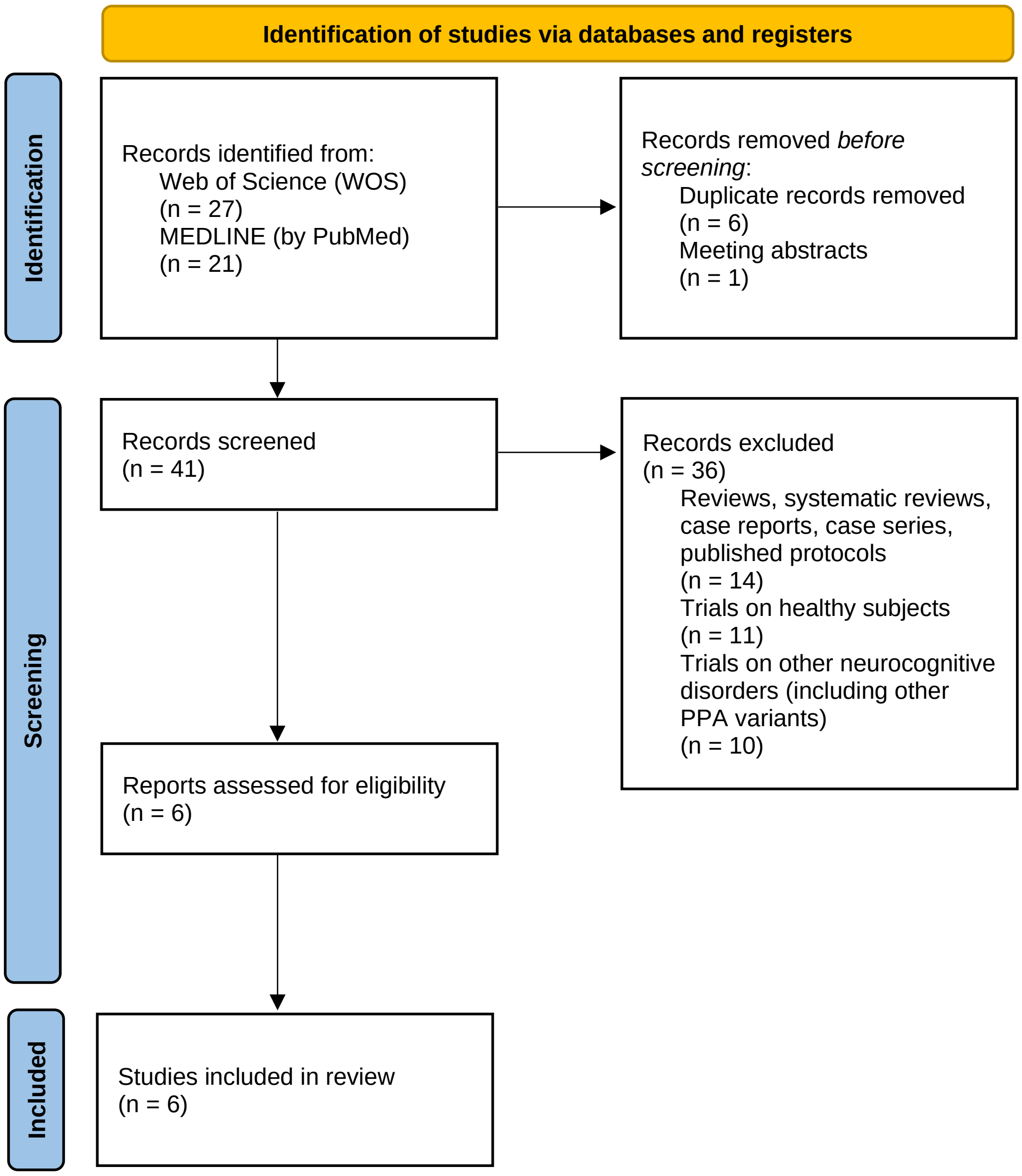

From: Page MJ, McKenzie JE, Bossuyt PM, Boutron I, Hoffmann TC, Mulrow CD, et al. The PRISMA 2020 statement: an updated guideline for reporting systematic reviews. BMJ 2021;372:n71. doi: 10.1136/bmj.n71

For more information, visit: http://www.prisma-statement.org/

Supplementary Figure 1. PRISMA 2020 flow diagram for new systematic reviews which included searches of databases and registers only
